# Assessing participation in a community-based health planning and services programme in Ghana

**DOI:** 10.1186/1472-6963-13-233

**Published:** 2013-06-26

**Authors:** Leonard Baatiema, Morten Skovdal, Susan Rifkin, Catherine Campbell

**Affiliations:** 1 Integrated Social Development Centre, Accra, Ghana; 2 Institute of Social Psychology, London School of Economics and Political Science, London, UK; 3 Save the Children, London, UK

**Keywords:** Programme Evaluation, Spider-grams, Community Participation, Primary Health Care, Health Planning, Ghana

## Abstract

**Background:**

Community participation is increasingly seen as a pre-requisite for successful health service uptake. It is notoriously difficult to assess participation and little has been done to advance tools for the assessment of community participation. In this paper we illustrate an approach that combines a ‘social psychology of participation’ (theory) with ‘spider-grams’ (method) to assess participation and apply it to a Community-based Health Planning and Services (CHPS) programme in rural Ghana.

**Methods:**

We draw on data from 17 individual in-depth interviews, two focus group discussions and a community conversation with a mix of service users, providers and community health committee members. It was during the community conversation that stakeholders collectively evaluated community participation in the CHPS programme and drew up a spider-gram.

**Results:**

Thematic analysis of our data shows that participation was sustained through the recognition and use of community resources, CHPS integration with pre-existing community structures, and alignment of CHPS services with community interests. However, male dominance and didactic community leadership and management styles undermined real opportunities for broad-based community empowerment, particularly of women, young people and marginalised men.

**Conclusion:**

We conclude that combining the ‘spider-gram’ tool and the ‘social psychology of participation’ framework provide health professionals with a useful starting point for assessing community participation and developing recommendations for more participatory and empowering health care programmes.

## Background

Global health systems continue to be championed by biomedical scientists and health experts whose technocratic solutions to ill health provide community members with few opportunities to appropriate these solutions to local realities [1]. This tendency was challenged by the 1978 Alma Ata Declaration which established community participation as a core principle of primary health care [2]. Despite the revolutionary significance of the Alma Ata Declaration in viewing primary health care through the lenses of equity, social justice, and participation, shifts favouring community participation in international health policy have been slow and saw a decline in the late 1980s and 1990s [3]. More recent efforts however, spearheaded by the 2008 Lancet special edition to celebrate the 30 year anniversary of Alma Ata [4] and the 2008 WHO report on Social Determinants of Health [5], have revitalised the message that community participation is key to the delivery of health care. Many countries, including Ghana through its Community-based Health Planning and Services (CHPS) Programme, have since taken active steps to involve community members in addressing health problems at the community-level [6].

Alongside these efforts, much work has been done to conceptualise the pathways through which community participation might increase access to health services, improve health outcomes and promote health enhancing behaviours [7-9]. Despite a growing interest in ‘evidence-based public health’ and the proliferation of theoretical literature into community participation, there remains a dearth of tools and indicators for evaluating how communities participate in and influence programmes in practice. So whilst research exploring the community response to local health services has importantly focused on the impact or outcomes of their participation, measured in terms of factors such as changes in knowledge, attitudes and behaviour, few efforts have been made to explore how the process of community participation can be assessed and lead to changes [10]. Relatedly, there is a lack of evaluations that have examined local stakeholders’ own perspectives of their participation.

In this study we will contribute to this lack of knowledge in two ways. Firstly, we will present and explore the application of a combined theoretical framework and methodological tool to assess community participation. Secondly, we will explore the participatory processes of a community-based health planning and services programme in Ghana and argue that a narrow focus on programme outcomes ignores the extent to which programmes impact the sense of health-related agency of community members, enabling them to maximise the effectiveness of health programmes.

### The community-based health planning and services programme of Ghana

Inspired by the Alma Ata commitment to primary health care, and regarded as a bold departure from bureaucratic models of health service delivery, CHPS is a national health policy initiative that was adopted in 1999 [11]. The initiative, as a strategy to increase rural access to health care service while empowering local communities to take greater control over their health, sought to promote community-driven health care services, with technical support from the central Ghana Health Service. The CHPS initiative is an extension of the Navrongo experiment (pilot project of CHPS). Initiated in 1994, it advanced the idea that the mobilisation of traditional systems of leadership, resources, communication and governance had the potential of increasing health-care services accessibility, reducing child and maternal mortality whilst improving rural-population’s overall health (ibid.). At a National Health Forum in 1999, the Ghana Health Service disseminated results from the Navrongo experiment and subsequently drafted a policy statement coining the acronym and legitimizing CHPS as a national community health care initiative with the Ghana Health Service (GHS) assuming oversight responsibility.

The CHPS strategy advocates the systematic planning and implementation of primary health care facilities^a^ and activities with active participation of community leaders and members. In practice, this is achieved through the mobilization of community leadership, decision making systems and resources in within defined catchment areas (zones) [12]. CHPS is integral in other Ghanaian government policy agendas including the current National Health Policy. It has been cited as a major healthcare care reform strategy in Africa with health services adapting to local needs and circumstances [11]. In this paper we draw on a social psychological understanding of participation and the spider-gram method to assess community members’ experiences of participation in this promising programme and explore what more can be done to make programmes of this nature more participatory.

### Assessing participation in community-based health care programmes

Much scholarly work has highlighted the difficulties of evaluating community participation in health interventions [2,13,14]. With growing interest and pressure to involve local communities in global health practice, there is a pressing need to develop theoretical and methodological tools that assess the processes underlying participatory programmes. In this paper we explore how combining Campbell and Jovchelovitch’s [8] conceptualisation of a ‘Social Psychology of Participation’ and Rifkin et al’s [15] ‘Spider-gram’ can be used to assess participation in community-based health care programmes. We introduce each in turn.

### Theoretical framework: social psychology of participation

Much of the literature on community participation is driven by ideological and political commitments to participation, contested and framed either as a basic human right, a pragmatic strategy to utilise services or as pathway to empowerment [16]. In this paper we draw on the theoretical insights of a social psychology of participation, which leans towards the model of empowerment. The social psychology of community participation was promulgated by Campbell and Jovchelovitch [8] as a conceptual framework for action research seeking to explore the pathways between community participation, health and social development. The starting point of this framework is that the poor and marginalised often lack a sense of control over their health and well-being, leading to a sense of fatalism, and a tendency to wait for outside actors and agencies to take control of local health problems. Against this background, the framework seeks to draw attention to ways in which communities can be ‘empowered’ to exercise greater agency over their health, by changing health-damaging behaviours where possible, and making optimal use of available health services. Drawing on Habermas’ [17] idealised notion of the public sphere, the framework advocates that for participation to offer community empowerment, it should take place in a social space (public sphere) where all participants (in this case health service providers and users) have the right to participate fully in the design, implementation and evaluation of health programmes, with programmes being driven by a synthesis of ‘local’ and ‘expert’ knowledge, with both knowledge systems being accorded equal respect. Freire [18] suggests participation is most likely to empower marginalised communities to exercise greater control of their lives (and more specifically their health) if it is framed within a dialogical and facilitative approach through knowledge negotiation and power transfer from health professional to communities [19,20].

Health-enabling community participation should involve genuine sharing of power amongst health experts and decision makers on the one hand, and marginalised groups on the other [21]. Such an approach is said to build a sense of community ownership of local problems (as opposed to a sense that such problems can only be solved by outside professionals), and to encourage communities to contribute to the development of concrete strategies through which they can improve their health [22,23]. This approach resonates with the views of Robert Chambers who argues that poor communities can be empowered by taking responsibility and action in cases where experts are ready to share power and control over programs [24].

### Methodological tool: spider-grams

The Spider-gram methodology was developed by Rifkin et al., [15] to measure, visualise and locate levels of community participation in health programmes on a continuum. From an analysis of over 200 case studies [25], Rifkin and colleagues identified five indicators: *Needs assessment* refers to the roles played by programme beneficiaries in identifying their health needs and in designing the community intervention. L*eadership* emphasises the inclusiveness and representativeness of all community interests groups. *Organisation* refers to the extent to which new community interventions integrate or collaborate with pre-existing community structures or networks. *Resource mobilization* refers to communities’ ability to mobilise and contribute resources towards a community–based intervention. *Management* refers to community’s capacity to take decisions about the programmes’ direction and development.

Each indicator is located on a continuum. The original spider-gram plotted these indicators on a continuum that at one end marked narrow participation and at the other marked wide participation. This continuum was modified by Draper et al. [2] to place mobilization at one end and empowerment at the other. For this research the original continuum was used. The continua are linked together at the narrow end to form a pentagram. Each continuum is used to grade how wide or narrow community participation is. In a group setting, community members are asked to grade, from 1 to 5, the level of participation they felt was involved in the programme, with 1 reflecting a low level of participation and 5 reflecting the highest level of participation. To illustrate this, as well as to operationalize these indicators in relation to a continuum of participation, we have in Table 1 applied the principles of spider-grams to the CHPS programme.

**Table 1:** Indicators for spider-gram

**Indicators**	**Narrow, nothing**	**Restricted, small**	**Mean, fair**	**Open, very good**	**Wide, excellent**
	**1**	**2**	**3**	**4**	**5**
**Needs Assessment**	Identified or imposed by health experts without community involvement or consultation	CHPS services designed by health experts with limited community involvement	Community was consulted and involved in assessing their needs	Community involvement in needs assessment, and few services resonating with their assessed needs	Full community involvement in needs assessment with service package in resonance with their health needs
**Leadership**	Dominant-imposing CHPS committee chairman represents only committee or few elite or rich community members	Limited committee role in leadership, few representation of women or few interest groups	Few community consultation, involvement in decision-making and represent community interest	Good committee leadership role consults community, leadership constitute women representation and all interest groups	CHPS committee fully represents diverse interests, Selfless leadership roles, full community involvement in decision-making
**Organisation**	Parallel operation or no collaboration of CHPS with pre-existing community units or local structures	Limited collaboration of CHPS with pre-existing community units or structures	CHPS cooperates with few community structures	Integration and collaboration of CHPS with other community bodies	CHPS well and fully integrated and works collaboratively with other community units
**Resource Mobilization**	No community support or resource contribution. Community not involved or consulted in resource allocation	Limited amount of resources raised by the community. No community control over mobilised resources utilisation	Community raised resources and fully support CHPS with limited role in controlling expenditure	Community are resourceful and supports CHPS with mobilised resources. Community involved in resource allocation	Full and active community contributions to support CHPS. community fully consulted in resource allocation
**Management**	Managed or induced by service providers (GHS). No community consultation in management decision making	CHPS operation overseen by GHS with CHPS committee role	CHPS operation overseen solely by the health committee	CHPS committee self-managed and involved community and other interest groups (women) in decision making	Committee independently managed CHPS with full community consultation and representation

**Figure 1:**
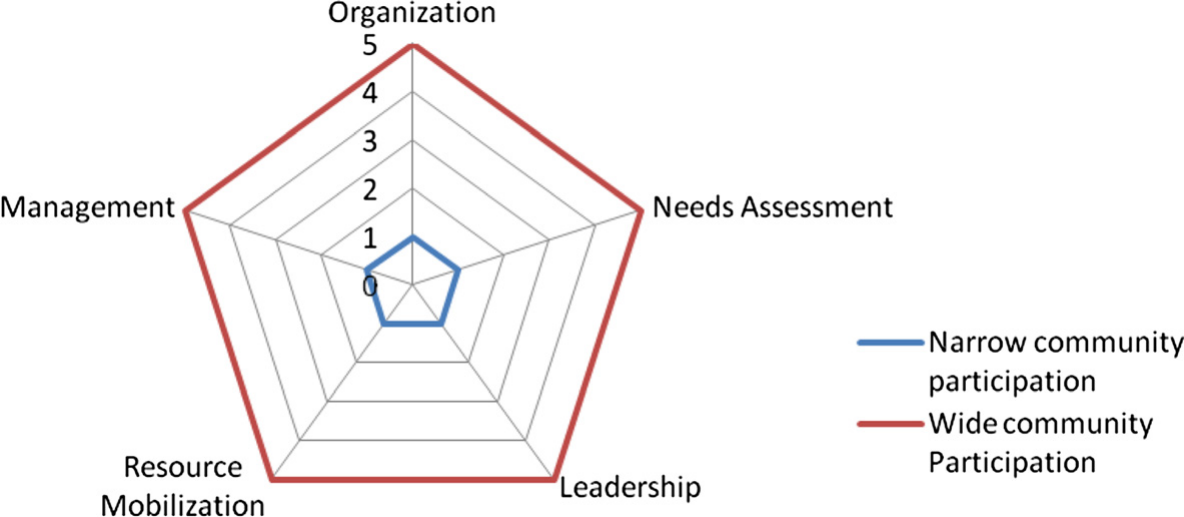
**Spider-gram for measuring community participation **[15]**.**

Spider-grams can illustrate levels of community participation as perceived by community members (see Figure 1) and be used to comparatively assess community participation across programmes, with different participants in the same programme as well as tracking to see changes in community level of participation in a particular programme over time. In view of its simplicity, applicability, and wide acceptance, it has been applied in many different contexts and studies [26-32].

## Methods

This qualitative study seeks to understand the processes underlying CHPS programme design and delivery, particularly in relation to social context, with the purpose of assessing the level of community participation that the programme enabled. Permission to conduct this study was granted by a research ethics committee at the London School of Economics and the Ghana Health Service. Verbal consent was formally sought from all informants.

### Study location and participants

The study was conducted in the Wa Municipal of the Upper West Region of Ghana, West Africa. The Municipality, according to the 2010 Population and Housing Census has a total population of 116,460. Waala is the main tribe in the Municipality although other ethnic and tribal groupings are resident in the municipal. In terms of healthcare delivery, there is a government hospital in the capital (Wa) of the Municipality and a few private clinics. Geographical accessibility to specialist health care is therefore not only difficult for the majority of people living in this part of the country but also skewed in favour of communities located within the Wa Municipal.

Optimising community access to healthcare and other social services in the region has been a long-standing challenge for the government of Ghana. Since the inception of the CHPS initiative in 1999, the government, with support from the Japan International Cooperation Agency, has been committed to scaling up CHPS facilities throughout the region. CHPS facilities are health care delivery centres, which are meant to be managed and run by the communities they serve. In this study we assess the level of participation by community members in the planning and running of the Nachanta CHPS health care facility, which is one of fourteen CHPS facilities in the Wa Municipality of the Upper West Region. Nachanta was purposively selected for this study primarily for being an averagely performing CHPS zone according to the Municipal Health Directorate and thus avoiding a skewed focus on either good or poorly performing CHPS facilities. Secondly, community members had participated in a discussion about CHPS on a local radio station and had therefore shown a previous interest in discussing their experiences of CHPS. The Nachanta CHPS opened in June 2008 and is jointly managed by three communities; Nakori, Tampieni and Chansa with a total population of 4,237 people. The community members are predominantly subsistence farmers cultivating crops such as maise, beans and rice. The Nachanta CHPS facility has been running for nearly three years at the time of the study.

To develop a holistic understanding of community participation in Nachanta CHPS, we examined the perspectives of three groups of stakeholders. We involved both service providers (n=3; 67% female, mean age of 52), users (n=12; 50% female, mean age of 46) and those at the interface, serving a role at the local community health committee (n=4; 50% female, mean age of 50). Service users in this study refer to adult community members from communities (Nakori, Tampieni and Chansa) within the Nachanta health centre catchment area. They were purposefully recruited [33], pages 169–186 through convenience sampling, based on their close proximity to the location of the interviews, interest and availability to participate. Service providers and community health committee members were recruited through criterion sampling, with the criteria either being the nature of their involvement in the provision of services or active participation in the local community health committee. Deliberate efforts were made to recruit a mix of male and female informants to explore the gender dimension of community participation.

### Data collection and analysis

Data were collected during two weeks of April, 2011. Before the data collection period, two preliminary visits were made to the study communities by the research assistants. The first visit was to sensitise the communities and meet with the opinion leaders to discuss the study and formally seek their consent and approval. Through this process, a rapport was established between leaders and research assistants. Issues of venue for the interviews, time period for the interviews, focus groups discussions and the community conversation were clarified in the second visit. The visits offered the researchers the opportunity to respond to questions posed by community members and also identified potential respondents. Data was collected by two research assistants in the local Waala language and supervised by the first author, all three of whom are local social science graduates with a wealth of experience in social research. Furthermore, they understood the local language and socio-cultural context of the study area. A quality assurance check list was developed to enable the first author to check data quality, including the translation and transcription of data.

As Table 2 indicates, data were collected in three different stages, with individual interviews and focus group discussions leading to a ‘community conversation’ [34] where all the study participants came together to discuss and draw up a spider-gram reflecting their consensus on the level of community participation that characterised the Nachanta CHPS. As a result of this step-by-step process, informants participated to varying degrees. For example, only two community health committee members participated in individual interviews. An additional two health committee members joined the focus group discussions, of which one did not have the time to subsequently participate in the community conversation.

**Table 2:** Distribution of research participants

**Data collection steps**	**Study participants**	**Sample**	**Characteristics**	**Sampling method**	**Purpose**
1. Individual interviews	Service providers	3	Chairperson of a community health committee	Criterion sampling	To get an insight into how they understand community participation and facilitated the programme accordingly.
			CHPS senior official		
(8-13 April, 2011)			Community health worker		
	Community health committee members	2	Community members involved in the local community health committee and at the interface between service providers and users.	Criterion sampling	To understand what community health committee members, who played a dual role, both as implementers of the CHPS programme and as beneficiaries, felt about their level of involvement.
	Community members (service users)	12	Community members making use of health services	Convenience sampling	As the programme was meant to involve the wider community, community members and service users were interviewed in order to examine their level of involvement in the programme.
2. Focus group discussions	Mix of female stakeholders	8	Service providers (0)	Criterion sampling	The focus group discussions were arranged to stimulate debate and develop responses as informants re-call and add to the answers of peers within the group
			Community health committee members (2)		
(14th April 2011)			Service users (6)	Convenience sampling	
	Mix of male stakeholders	9	Service providers (1)	Criterion sampling	
			Community health committee members (2)		
			Service users (6)	Convenience sampling	
3. Community conversation	All study participants	16	Service providers (1)	Criterion sampling	To bring local stakeholders together to discuss and develop a spider-gram assessing community participation in the CHPS programme
(14^th^ April, 2011)					
			Community health committee members (2)		
			Service users (6)	Convenience sampling	

We conducted a total of 17 in-depth individual interviews: 3 with service providers; 12 with service users; and 2 with community health committee members. Those who were individually interviewed were subsequently invited to participate in one of two focus group discussions made up of a mix of service users, providers and community health committee members to spark debate and enrich their responses in the process. The focus group discussions were segregated according to gender to ensure that weaker voices (most frequently women in this context) are not overshadowed by more powerful members of the group in the community (most frequently men). Two additional community health committee members joined the focus group discussions. The female focus group discussion consisted of 2 community health committee members and 6 service users, whilst the male focus group was made up of 1 service provider, 2 community health committee members and 6 service users (see Table 2).

**Figure 2:**
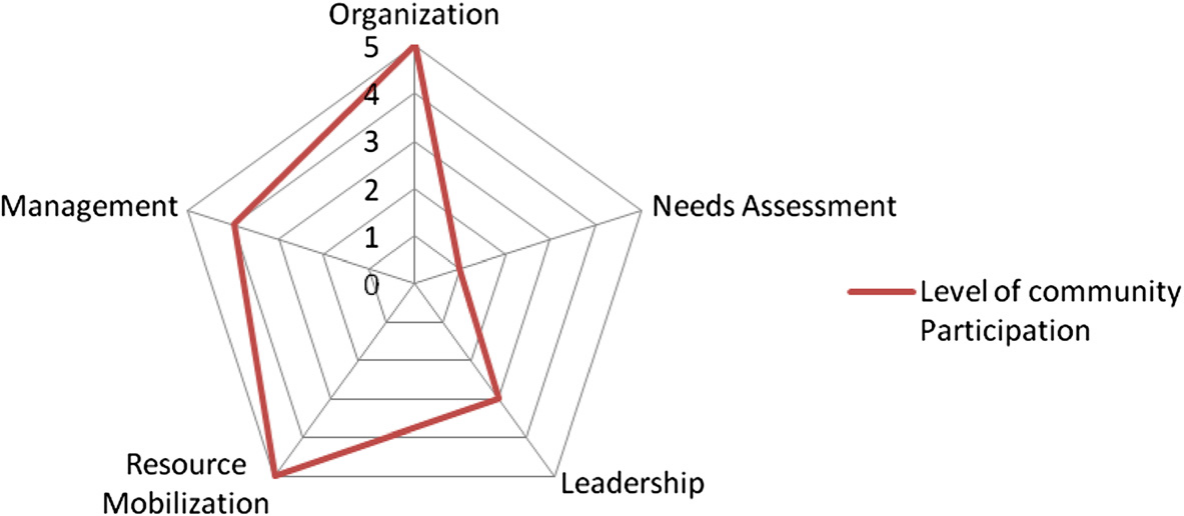
Level of community participation in Nachanta CHPS.

The interviews and focus group discussions were semi-structured and followed a topic guide with open-ended questions, which focused on the central research themes of needs assessment, management, organisation, resource mobilisation, and leadership. This allowed the researchers to both stay on topic whilst also having the flexibility to explore surprising but relevant responses further through prompts and follow-up questions. The two focus group discussions lasted between 115 and 130 minutes whilst individual interviews lasted between 50 and 60 minutes. After the interviews and the focus group discussions were completed, we invited all the informants to participate in a community conversation with the aim of collectively evaluating community participation in Nachanta CHPS. The spider-gram enabled a discussion and process to take place that culminated in a consensus, ranking each indicator of community participation a score as illustrated in Table 1 (see Table 3 and Figure 2 for the scores of the participants).

**Table 3:** Coding framework

**Codes from data**	**Basic themes**	**Organising themes**	**Global themes**	**Spider-gram scores**
**Barriers to community participation**			Needs Assessment	**1**
– Health team from Wa	– Non-involvement of communities in programme design	– CHPS was designed externally by health experts		
– We wanted a health centre				
– Programme designed outside the community	– Community meetings were limited to a few			
– Community not involved				
– Only unit committee chairman and a few were consulted				
**Facilitators of community participation**				
– We decided on CHPS site	– Community members chose CHPS site	– High decision making role		
– Community sensitised on about CHPS	– Community awareness about CHPS			
**Barriers to community participation**			Leadership	**3**
– committee was selected	– Undemocratic decision making processes	– Undemocratic leadership style		
– Chairman and the committee	– Vertical leadership style	– Patriarchal leadership		
– decisions made unilaterally by the committee	– Low female representation in committee			
– Don’t know about women role				
– women not in the committee				
**Facilitators of community participation**				
– Dedicated and hardworking	– High community confidence in committee	– Selfless and represent community interest		
– Represent all our interest				
**Facilitators of community participation**			Organization	**5**
– Working with health volunteers	– CHPS engage with community structures	– CHPS integrated well with pre-existing community structures		
– traditional birth attendants(TBA) give support	– CHPS tolerance with community networks			
– unit committee team support and engage with CHPS				
**Barriers to community participation**			Resource mobilisation	**5**
– Everyone is poor	– Resource MobilizationCommittee dominance in resource contributions and allocation	– Contribution not pro-poor		
– everyone contributes equally	– Controlled exclusively by committee	– Less community control		
– decisions made exclusively by health committee	– Internal resources	– Lack of external support		
– contributions given to committee				
– within community resources				
**Facilitators of community participation**				
– Supported and contributed fully	– Full community support for CHPS	– community actively contributed to support CHPS		
– CHPS maintenance	– Community highly resourceful			
– Contributed labour, bought stones, carried sand, water, etc.	– Contributions on gender lines			
– Contribute based on gender				
**Barriers to community participation**			Management	**4**
– We(females) are not involved	– ManagementNon-inclusiveness of management structures	– Less community influence and voice in management		
– Only the committee	– Ineffective management	– Limited management capacity		
– No skills training				
**Facilitators of community participation**				
– Committee not influenced in CHPS supervision and management	– Favourable management structures	– CHPS independently overseen by committee		
– Cordial relation with GHS	– Self-governing committee			
– Decision-making structures represent all interest groups				

As we sought to do a thematic analysis, transcripts of both individual interviews and focus group discussions were imported into Atlas.Ti, a qualitative software package, for coding. Through a process of reading and re-reading, data were categorised, giving rise to 168 codes, reflecting the wealth of themes emerging from the interviews. We do not seek to report on all of these codes, but following the thematic network analysis procedure as outlined by Attride-Stirling [35], identified codes from the larger pool of codes based on their recurrence and interconnectivity with the conceptual frameworks and the reviewed literature, a process that resulted in selection of 32 codes. In the second step, basic themes were explored and both facilitating and barriers to community participation themes were noted, paying particular attention to the different views of our three groups of study participants. These were further examined into higher organising themes (step three). In step four, five pre-determined themes constituted our global themes. At this stage, the global themes were categorised according to their resonance with the predetermined thematic areas suggested by Rifkin et al. [15]. Table 3 depicts the coding framework and illustrates how the themes are connected as we moved from codes to basic, organising and global themes. We use the coding framework to structure our discussion of findings.

## Results

In this section we present the views and perspectives of our informants, representing three groups of programme stakeholders: 1) community health committee members, who were given significant responsibility over the operations and implementation of the CHPS programme, and consisted of two male community opinion leaders as well as two female ‘magazias’, elderly female leaders who act in the interest of other women. They were elected for the committee role by local chiefs and other community opinion leaders, with no consultation done with the wider community; 2) Service users were made up of community members from the three local communities. They were largely poor and unaware of the work that was done to set up the health facility, and thus did not participate in the decision making about how it was run, although, as discussed below, they were occasionally called on to help in specific circumstances where labour or small donations were needed; 3) Service providers participating in the study were by our other informants referred to as health experts and health personnel from the Wa Municipal Health Services.

In the process of explaining (individual interviews), discussing (focus group discussions) and collectively evaluating (community conversation) community participation in the CHPS programme, different views and perspectives, pertaining to how community participation was applied in the programme, were inevitably raised. Although our three groups of informants eventually came to a collective consensus, indicating both wide and narrow community participation as illustrated by the spider-gram in Figure 2, our material is characterised by differing views – some of which we now seek to tease out.

### Needs assessment

From the interviews, the view shared by all participants was that the community did not play any role in identifying their health needs or designing CHPS. From their accounts, the program was designed by health experts from Wa without their inputs or participation. Despite this pattern, the study revealed community members welcomed and supported the program because it significantly resonated with their health needs. The excerpt below is a male participant’s reflection on this.

*‘….No! We don’t have any idea how this was done. The team didn’t even do any needs assessment; they brought us CHPS and this is good. We wanted a health centre and not CHPS but it is good that we have been given this because it serves our health needs but not everything’ (Male service user in individual interview* )

Contrary to the dominant view that their needs had been pre-defined by the service providers, a minority of participants indicated there were indeed community consultations and meetings about the CHPS program before it was brought to them. This was echoed further by interviews from the service providers namely the Municipal CHPS coordinator and the community health committee chairman. The chairman reported they held three meetings with them on issues of implementation and these were discussed and agreed by all during the community meetings before the start of the CHPS implementation. He intimated the meetings were organised for community members to reach a consensus on how the programme could well be implemented. Below is an expert from the committee chairman in this regard.

‘Well! We were part of the whole process from the very beginning. The community met three times to plan and discuss how CHPS implementation will be executed – this happened immediately the health team informed us about the CHPS programme and the roles we were required to play. Community members’ attendance in these meetings was not encouraging at the initial planning stages but subsequently the numbers increased and the support base for community implementation of CHPS increased and this explains why CHPS is been successfully implemented here’(Male health committee chairperson in individual interview)

In short, service providers and some service users appeared to be more aware of opportunities for participation than other service users. However, in reaching a consensus on their level of participation, service users rated their participation in needs assessment at point-1 on the spider-gram indicating very low level of participation.

### Leadership

Under leadership, different views were expressed by both service users and service providers regarding the leadership style and composition of the community health committee. Interviews from majority of the service providers revealed that the leadership style and composition of the community health committee represented all interest groups and that its activities and decisions served their interests and not that of the committee or any individual. Few service users held contrary views to this. Affirming the trust community members had of the community health committee’s leadership role, a participant ran the following commentary:

‘…No! I know anything they decide or undertake is best for us. They know our situation. The committee works selflessly for the interest of everybody and I have no reason to doubt the agenda behind their work’ (Male service user in individual interview)

However, all informants were unanimous that programme leadership was completely male dominated.

‘We don’t take part in community meetings about CHPS, our husbands do. When it has to do with contributions to support CHPS, then our services are needed. This is the case in this community like any other community around here but there is nothing we can do because that is how life is structured in this setting and it has always been so’. (female service user in focus group discussion)

Another informant commented rhetorically on the complete absence of female representation in the CHPS process:

‘…*assemblyman, assemblyman, are there even women in that your committee? I mean the committee in charge of CHPS in the community*?’ (*Male service user in focus group discussion*)

The community health committee has decision making powers with less community involvement. Community members only get involved when decisions have been made and they get informed of the roles expected of them. Following deliberations on where to peg their role in leadership, point 3 of the spider-gram was reached by service users.

### Resource mobilisation

Findings from the interviews revealed the communities had made significant ‘in kind’ and ‘cash’ contributions to support the program. Contributions in kind took the form of water, sand, purchase of stones, among others for the construction and maintenance of CHPS, as well as the labour to build and sustain the buildings. Contributions were also made in cash to support the building and maintenance of the CHPS program.

‘We fetched water, carried sand, we really did a lot. Will there be such a project without payment? We are even fed up with the contribution to maintain this compound. We did a lot of contribution from the very start until the end and even now, we still occasionally contribute to repair broken parts. Go and have a look around and you will be marveled at what these small and poor communities like ours have been able to achieve. The building alone speaks of what contribution we did.’ (Female service user in individual interview)

Our findings suggested that contributions to labour and materials were made by a wide range of community members irrespective of their economic status or gender. However, inputs were gender differentiated, with men and women. The men contributions were in the form of labour, cash, digging of sand, etc. whilst the women carried water, sand, cleaning the surroundings of CHPS.

*Madam Fati! You know everybody’s situation in these communities. We are all poor. Once you are living in a community like this, you are considered poor or else you would be living around Wa or the Kambali town areas or the Xavier community. We all share the same problems and so no one is higher, lower or expected to be given any preferential treatment. But contribution was segregated by gender. You know we can’t contribute equally, we have our share to contribute and our male counterparts have theirs. But for us, we all contribute equally; if you don’t, your fellow women will discount you’.* (*Female service user in focus group discussion*)

Also, service users said that decisions on contributions were exclusively discharged by the community health committee, that is, decisions were vertically made. The community health committee had absolute and unquestionable decision making powers over what needed to be contributed. However, community members shared the view that their support of the programme has empowered them. Corroborating with data elicited from the in-depth interviews, the two focus groups affirmed that their contribution and support to implement CHPS has been tremendous and as a result they firmly rated the community’s contribution on the spider-gram at point 5.

### Management

The findings showed that CHPS was independently managed by the local community health committee without any outsider influence. Some service users in the interviews also spoke and expressed confidence in how the community health committee was managing and overseeing the CHPS programme implementation. A participant highlighted this point in the following extract:

*‘…Certainly! The committee is managing the programme very well without external or outside (GHS) influence. We might be small at the moment and not representing all but this is a view widely held in these three communities if you want to enquire further to establish the fact’.* (*Female service user in focus group discussion)*

Also, the study indicated that management and decision making structures were vested under the authority of the community health committee who made decisions vertically without full community members’ engagement. Community members (service users) were only at the receiving end of decisions unilaterally made by the community health committee.

***‘****Hmmm****…****what can we say since we are not them? I mean the committee. Ok. From outside point of view, I think everything is working alright without external interference. We would have been made aware if there were such issues’. (Male service user in individual interview)*

Again, female representation in the management structures was silent as findings indicate a male-dominated management style. The service users when prompted about their view on such an arrangement did not have any reservation about it. They appeared to be satisfied with what they regarded as the good management roles and decision making structures coordinated by the committee. Regarding opportunities for management capacity building, the committee chairman indicated that they had no access to capacity and skills training programmes to enhance their capacity to manage the programme. On the whole, in rating their participation in managing the CHPS on the spider-gram, majority of service users solidly agreed on point-4 as the extent to which they participate in the program although this did not fully reflect the positions held by all services users as some few service users held contrary views regarding their level of participation in managing CHPS.

### Organization

Findings from the study revealed that the CHPS programme successfully integrated itself into pre- existing community structures that predated the establishment of the CHPS. Some of these structures that had existed in the community prior to the advent of the CHPS programme the study revealed include a unit committee, health volunteers and traditional birth attendants. The study gathered that all these community structures were all absorbed and fully integrated into the CHPS programme in order to avert any confrontation or conflict between CHPS and the community structures. In highlighting on the degree of CHPS integration with the community structures, a health committee member gave the following commentary:

‘The unit committee and the volunteers are still very active and working to support the CHPS as I said. Some of the unit committee members are playing dual roles as unit committee members and community health committee members. I was a member of the unit community when the programme started and from experience I know although they are parallel structures, they work collaboratively’. (Male service provider in individual interview)

Following this, participants deliberated on where to rate their level of community participation on the spider-gram. Subsequently, participants unanimously agreed that pre-existing community structures were fully integrated into the CHPS programme and so they rated it at point 5.

## Discussion

In light of earlier assertions regarding the dearth of workable analytical tools to assess community participation [36,37], we set out to explore the potential of combining spider-grams and a social psychological understanding of participation to assess levels of community participation in a CHPS programme in Ghana.

Through this method and theory combination we have been able to highlight a range of factors impacting optimal and empowering community participation. Some of the factors facilitating community participation included community mobilisation of local resources to support CHPS (communities made significant ‘in kind’ and ‘cash’ contributions to support the program), CHPS integration with pre-existing community structures (existing unit committees, health volunteers and traditional birth attendants were all absorbed and fully integrated into the CHPS program), representativeness of community interests and working independently without external interference from health professionals. Factors hindering community participation included top-down approach to CHPS design, male dominance and vertical-undemocratic community leadership and management styles. It appeared that management and decision making structures were vested under the authority of the community health committee who made decisions vertically without full community members’ engagement, an arrangement which disempowered community members.

What opportunities for participation did the CHPS programme offer potential service users in Nachanta? Our findings suggest that the programme was largely imposed on the community by outside experts - externally designed with limited community participation. Such a situation would be regarded as inimical to optimal community empowerment, with theorists such as Wallerstein [38], Rappaport [19] and Laverack [20] arguing that programmes seeking to promote ‘empowerment via participation’ should involve the active participation of all community members in order to offer opportunities for acquisition of knowledge and skills, confidence, personal experiences of efficacy, ability to identify and solve one’s problems.

Examining our data in a social psychological understanding of participation [8] enabled us to identify the limitations of implementing community participation in a way that facilitated community empowerment. These limitations highlighted a technocratic conceptualization of community participation resulting in domination by non-elected leaders, male dominance and vertical decision-making. In this regard, for all its advantages, the project did not meet the ideals of equal participation of all players irrespective of their gender, age or social status, with all players being fully consulted at every stage of programme design and implementation, and with the knowledge and views of each group carrying equal weight in these processes. In this regard the project missed out on valuable opportunities to increase the confidence and ability of the most marginalised project stakeholders to take control over their lives and their health.

These observations corroborate the argument of Rifkin [25] and Goodman et al. [39] which claims that the structure of community leadership is often historically or culturally determined to exclude marginalised groups including women, young people and marginalised men, with such social exclusion being widely regarded as a contributor to the health inequalities often suffered by such groups [5].

For community participation to be effective and empowering there is an overarching need for health planners and programme developers to engage with rank-and-file community members in dialogue about service provision, where local knowledge is taken as seriously as expert knowledge. Whilst external health experts, change agents or facilitators have specific technical knowledge that can aid participation, such technical expertise must be accompanied with local knowledge (e.g., resources, culture, gender and power relations), requiring local people to participate. Using social psychology as part of a framework to examine participation highlights how failure to recognise the importance of involving local people and local knowledge challenges the alignment of health services with local realities and runs the risk of health services simply reproducing the status quo of experts definition. It also charts the course of health programmes in marginalised communities that restricts their roles in decision-making and opportunities to gain knowledge, skills and confidence to become seriously involved in the direction of health programs. In our study a limited number of respondents mentioned such participation. They tended to express a sense of passivity and distance in relation to the control of CHPS. Opportunities for participation were limited to what Arnstein [40] would describe as tokenistic (form of community participation where community members are only informed or consulted purposely to seek their consent) offering reduced opportunities for enhancing community members’ sense of agency.

We also found women to have fewer opportunities to participate than men. The male-dominated nature of the participation that did occur was a predominant theme in our findings, particularly in relation to the theme of resource mobilisation, leadership and management. ‘It is the committee’; ‘them’; ‘they will know’; ‘we don’t contribute equally to support the program’; and ‘the committee’ were constant phrases heard from women in connection with decision making relating to all three themes. Our study highlights how effective community participation can be hampered by relational factors, such as those that concern gender. It also calls for further studies to unearth and bring to the fore the relationship between gender and health in the contexts of CHPS in Ghana or other similar contexts.

Differing views of how community participation played out in practice were evident. Community members (service users) saw their participation in the CHPS programme as limited. They were not involved in the design process, only being asked to contribute in cash or in kind to maintain the CHPS compound – merely reflecting the utilitarian value of community members in health programme implementation. They saw CHPS leadership at the community level as one imposed on them as they played no role in selecting committee members. Service providers and community health committee members on the other hand believed that community members (service users) were adequately consulted before the commencement of the programme, and did have a role to play in electing the community health committee as well as managing the CHPS programme. These conflicting understandings of how community participation played out, underlining the contested nature of community participation, have been noted elsewhere [2,41]. These differences are arguably rooted in the reluctance of more powerful actors to relinquish their power and control and the desire of community members to be more involved in services and decisions that impact their health and well-being. This, coupled with the reality that health professionals, in virtue of their expert knowledge, will always be in a position of power, further complicates genuine power-sharing in participatory health programmes.

Whilst local people played a key role in providing resources (materials and labour) for the building and maintenance of the health centre, reflecting their utilitarian value, capacity building was limited, evidenced by their struggles to maintain and repair the facility. Whilst it is important for community members to contribute with resources, engaging in a community-government partnership that bolsters community ownership, this partnership needs to have a long-term strategy, with funding agencies committing to avail resources as and when required to sustain the health initiative and by building the capacity of community members and groups, enabling them to develop the programme and seek alternative sources of funding. Such a strategy responds to concerns raised by Hill [42] who argues that it is often the most economically unsound communities that are required to mobilise resources to support their health when access to health should be their fundamental human right. The lack of a long-term strategy of the Ghanaian CHPS programme runs the danger of creating a context where poor communities are compelled to contribute to the health initiative and failure to contribute could result in them being denied access to health care. There is a need for external support agencies to recognise their responsibility to sustain community-based health initiatives in the long-term and to capacitate community members and groups so that they can more easily link up and partner with resourceful organisations for joint ventures.

A key limitation of this study is the fact that it only reports on the experiences of one CHPS community. Being a small qualitative study, the observations presented in this paper are based on the subjective views and personal experiences of only a few informants and not the whole community. It is therefore difficult for us to generalise and comment on the CHPS programme as a whole. A second limitation relates to reporting bias, particularly by service providers, who have an interest in representing the programme in a positive light. A third limitation pertains to the weight given to spider-gram indicators. It is unclear whether a hierarchy exist regarding the importance and level of influence of each indicator in the spider-gram. This is an area for future research.

## Conclusion

Notwithstanding these limitations, we believe that insights like those presented in this paper are critical to translating policy rhetoric regarding community participation into practice. We conclude that spider-grams, plotted in group or community settings, framed and guided by a social psychology of participation, is a useful strategy for health professionals and community members alike to critically track and check that every step of the programme design and evaluation process is participatory. We believe that this method (spider-gram) and theory (social psychology of participation) combination is key to both assessing community participation and simultaneously encourage the agency of participants for more participatory community-led intervention.

## Endnotes

^a^The health care facilities have been referred to as CHPS compounds. These compounds serve as the centre for community health care services, such as outreach clinics for childhood immunization, health education activities, family planning services etc.

## Competing interests

The authors declare that they have no competing interests.

## Authors’ contributions

All authors were involved in ongoing discussions of the study design. LB managed the data collection, conducted the data analysis and wrote the first draft of the paper. MS, CC and SR provided supervision and contributed to the writing of the final draft. All authors have read and approved the final manuscript.

## Pre-publication history

The pre-publication history for this paper can be accessed here:

http://www.biomedcentral.com/1472-6963/13/233/prepub
